# Rapid and sensitive amplicon-based genome sequencing of SARS-CoV-2

**DOI:** 10.3389/fmicb.2022.876085

**Published:** 2022-08-17

**Authors:** Changwoo Park, Kwan Woo Kim, Dongju Park, Zohaib ul Hassan, Edmond Changkyun Park, Chang-Seop Lee, MD Tazikur Rahman, Hana Yi, Seil Kim

**Affiliations:** ^1^Microbiological Analysis Team, Group for Biometrology, Korea Research Institute of Standards and Science (KRISS), Daejeon, South Korea; ^2^Convergent Research Center for Emerging Virus Infection, Korea Research Institute of Chemical Technology (KRICT), Daejeon, South Korea; ^3^Department of Agricultural Biotechnology, Seoul National University, Seoul, South Korea; ^4^Department of Public Health Sciences, Graduate School, Korea University, Seoul, South Korea; ^5^Interdisciplinary Program in Precision Public Health, Korea University, Seoul, South Korea; ^6^Department of Biological Science, Chungnam National University College of Bioscience and Biotechnology, Daejeon, South Korea; ^7^Department of Bio-Analysis Science, University of Science and Technology (UST), Daejeon, South Korea; ^8^Research Center for Bioconvergence Analysis, Korea Basic Science Institute, Cheongju, South Korea; ^9^Department of Internal Medicine, Jeonbuk National University Medical School, Jeonju, South Korea; ^10^Research Institute of Clinical Medicine of Jeonbuk National University – Biomedical Research Institute of Jeonbuk National University Hospital, Jeonju, South Korea; ^11^Department of Medical Science, Jeonbuk National University Medical School, Jeonju, South Korea; ^12^School of Biosystems and Biomedical Sciences, Korea University, Seoul, South Korea

**Keywords:** SARS-CoV-2, reverse transcription digital droplet PCR, amplicon-based genome sequencing, MinION, long amplicon

## Abstract

As SARS-CoV-2 variants of concern emerged, the genome sequencing of SARS-CoV-2 strains became more important. In this study, SARS-CoV-2 was sequenced using amplicon-based genome sequencing with MinION. The primer panel used in this study consisted of only 11 primer panels and the size of the amplicons was approximately 3 kb. Full genome sequences were obtained with a hundred copies of the SARS-CoV-2 genome, and 92.33% and 75.39% of the genome sequences were obtained with 10 copies of the SARS-CoV-2 genome. The few differences in nucleotide sequences originated from mutations in laboratory cultures and/or mixed nucleotide sequences. The quantification of the SARS-CoV-2 genomic RNA was done using RT-ddPCR methods, and the level of LoD indicated that this sequencing method can be used for any RT-qPCR positive clinical sample. The sequencing results of the SARS-CoV-2 variants and clinical samples showed that our methods were very reliable. The genome sequences of five individual clinical samples were almost identical, and the analysis of the sequence variance showed that most of these nucleotide substitutions were observed in the genome sequences of the other clinical samples, indicating this amplicon-based whole-genome sequencing method can be used in various clinical fields.

## Introduction

A rapid and accurate diagnosis of SARS-CoV-2 is of the greatest importance in the current global pandemic. Although multiple RT-qPCR assays have been established and widely used for the detection of SARS-CoV-2, the increasing number of variants of concern is becoming a significant threat to the global health (Jung et al., 2020; Park et al., 2021; Yoo et al., 2021). Recently, many variants of SARS-CoV-2 have been reported, and four SARS-CoV-2 variants of concern (VOC) were labeled by the WHO. In December 2020, the Alpha (B.1.1.7) and Beta (B.1.351) variants were first recognized as VOC in the United Kingdom and South Africa, respectively. In January and March 2021, Gamma (P.1) and Delta (B.1.617.2) variants were initially identified from Brazil and India, respectively (CDC, 2021). A new emerging variant was reported from South Africa in late 2021 and the emerged variants were designated as new VOC (Omicron; BA.1, and BA.2) on 26 November 2021, and became new dominant variants (Kandeel et al., 2021).

The first genomic sequence of SARS-CoV-2 was released on 10th January, and more than 2.6 million genome sequences have been released by the Global Initiative for Sharing All Influenza Data (GISAID) to date (Elbe and Buckland-Merrett, 2017). Most of the genome sequence analyses of the SARS-CoV-2 clinical samples were done using NGS (Castillo et al., 2020; Stefanelli et al., 2020; Wang et al., 2020). WGS technologies can sequence millions of reads per run simultaneously with massively parallel processing and offers a greater discovery ability to detect rare or novel variants with deep sequencing (Maurier et al., 2019). However, the genome coverage can be low in a high-level background environment with a low concentration of virus (Quick et al., 2017). In some studies, genome sequencing based on multiplex PCR was performed from viral RNAs (Quick et al., 2017; Gohl et al., 2020; Kim et al., 2021). This protocol can enrich a small amount of the target gene, such as clinical samples. In addition, tiling PCR based on this protocol can reduce the number of tubes required for PCR and the experimental steps, enabling a faster genome sequencing. However, previous tiling PCR methods for viral genome sequencing produced short amplicons which were not efficient for long-read sequencers such as MinION because short amplicons require a large number of primer pairs for whole-genome sequencing, the primer mixtures become complicated especially for a large viral genome such as coronaviruses.

In this study, the viral RNAs from five clinical samples and cultured SARS-CoV-2 variants were quantified with a reverse transcription digital droplet PCR (RT-ddPCR) and sequenced as whole-genomes using a MinION sequencer with only 11 amplicons. The assemblies of the reads of the viral RNA amplicons showed high-quality genome sequences, which were almost identical to the assemblies without amplification using an Illumina sequencer (iSeq100), indicating that our methods can be used for the SARS-CoV-2 genome sequencing from clinical samples.

## Materials and methods

### Clinical samples and SARS-CoV-2 genomic RNA

The clinical samples used in this study were the same clinical samples used in our previous study (Park et al., 2021). The samples were collected from subjects according to registered protocols approved by the Institutional Review Board (IRB) of Jeonbuk National University Hospital with all patients having signed written informed consent forms (IRB registration number: CUH 2020–02–050-162,008). The clinical characteristics of the patients are shown in Supplementary Table S1 (Park et al., 2021). Upper respiratory tract specimens (naso- and oro-pharyngeal swabs) from COVID-19 patients were suspended in a transport medium (eNAT; COPAN, United States) and stored at 80°C until use. The RNA extraction of the clinical samples was performed using a viral RNA Minikit (QIAGEN, United States). The genomic RNAs of SARS-CoV-2 variants were obtained from the National Culture Collection for Pathogens (NCCP, Korea). The strains of the SARS-CoV-2 variants used in this study are listed in Supplementary Table S2.

### Viral genome sequences and primer design

The complete genome sequences of SARS-CoV-2 were derived from GISAID on 25 June 2020 (Elbe and Buckland-Merrett, 2017). The genome sequences with ambiguous bases were removed using PRINSEQ (Schmieder and Edwards, 2011). The number of trimmed sequences was 3,323, and the alignment of the genome sequences was done with MAFFT using the default option (Nakamura et al., 2018). The primers for the whole-genome amplifications were designed from conservative regions of the SARS-CoV-2 genomes.

The criteria for the primers were as follows: amplicon length, 2.7–3.4 kb; Tm, 53°C–61°C; primer length, 18–23 nt; overlap length between amplicons >150 bp; and number of degenerate bases <1. The specificity of the primer pairs was checked *in silico* using basic local alignment search tool (BLAST) against the GenBank Nucleotide (nr/nt) database. The results show that the primer panels were specific to the SARS-CoV-2 sequences. The size of the PCR amplicons was checked with agarose gel electrophoresis. The sequences of the primer-probe sets for RT-qPCR and RT-ddPCR are listed in Supplementary Table S3. The primer-probe sets were synthesized for RT-qPCR and RT-ddPCR by NeoProbe (Korea). All probes were labeled with the fluorescence of 6-carboxyfluorescein (FAM) at the 5′-end and Black Hole Quencher 1 (BHQ-1) at the 3′-end (Supplementary Table S3).

### Quantification of viral RNA

The extracted viral RNAs were initially quantified using the QuantiFluor RNA System (Promega, United States) and Quantus™ Fluorometer (Promega, United States). Thus, 1 μl of RNA templates was added to 200 μl of the QuantiFluor RNA Dye working solution, and the concentration was measured. The RNAs were serially diluted for reverse transcription-quantitative polymerase chain reaction (RT-qPCR) and reverse transcription droplet digital PCR (RT-ddPCR).

The RT-qPCR assay was performed using StepOne and the StepOnePlus Real-Time PCR system (Thermo Fisher Scientific, United States) with the One Step PrimeScript RT-PCR Kit (Perfect Real Time; TaKaRa, Korea). The total volume of the RT-qPCR reaction mixture was 20 μl, and the reaction mixture was prepared according to the manufacturer’s instructions. The RT-qPCR was carried out under the following conditions: reverse transcription at 42°C for 5 min, enzyme activation at 95°C for 10 min followed by 40 cycles of denaturation at 95°C for 10 s and annealing and extension at 60°C for 35 s (Supplementary Table S4).

The RT-ddPCR experiment was performed using the QX200 system (Bio-Rad Laboratories, United States) with a supermix for the probes (Bio-Rad Laboratories, United States). The total volume of the reaction mixture was 20 μl, and the reaction mixture was prepared according to the manufacturer’s instructions. The RT-ddPCR was carried out under the following conditions: reverse transcription at 42°C for 60 min, enzyme activation at 95°C for 10 min followed by 70 cycles with a 20% ramp rate of denaturation at 95°C for 30 s, and annealing and extension at 60°C for 150 s with a final enzyme deactivation at 98°C for 10 min (Supplementary Table S4). The copy numbers of viral RNA were determined according to a previous study and the manufacturer’s instructions (Bio-Rad Laboratories, 2015; Park et al., 2021).

### cDNA synthesis from the RNA samples

The cDNAs were synthesized from the RNAs extracted from the clinical samples, and the RNAs were obtained from NCCP using the LunaScript RT SuperMix Kit (NEB, United States). To assess the Limit of Detection (LoD) of the MinION sequencing, the extracted RNA of NCCP 43381, 43,382, 43,388, and 43,390 (NCCP VOC variants) was diluted to a concentration of approximately 10 copies/μl. The total volume of the reaction mixture was 20 μl and consisted of 1 μl RNA template, 4 μl 5X LunaScript RT supermix, and 15 μl distilled water. The reverse transcription reaction was carried out under the following conditions: primer annealing at 25°C for 2 min, cDNA synthesis at 55°C for 30 min, and heat inactivation at 95°C for 1 min. The synthesized cDNAs were used as templates for PCR without purification.

### PCR amplification for whole genome sequencing

**Figure 1:**
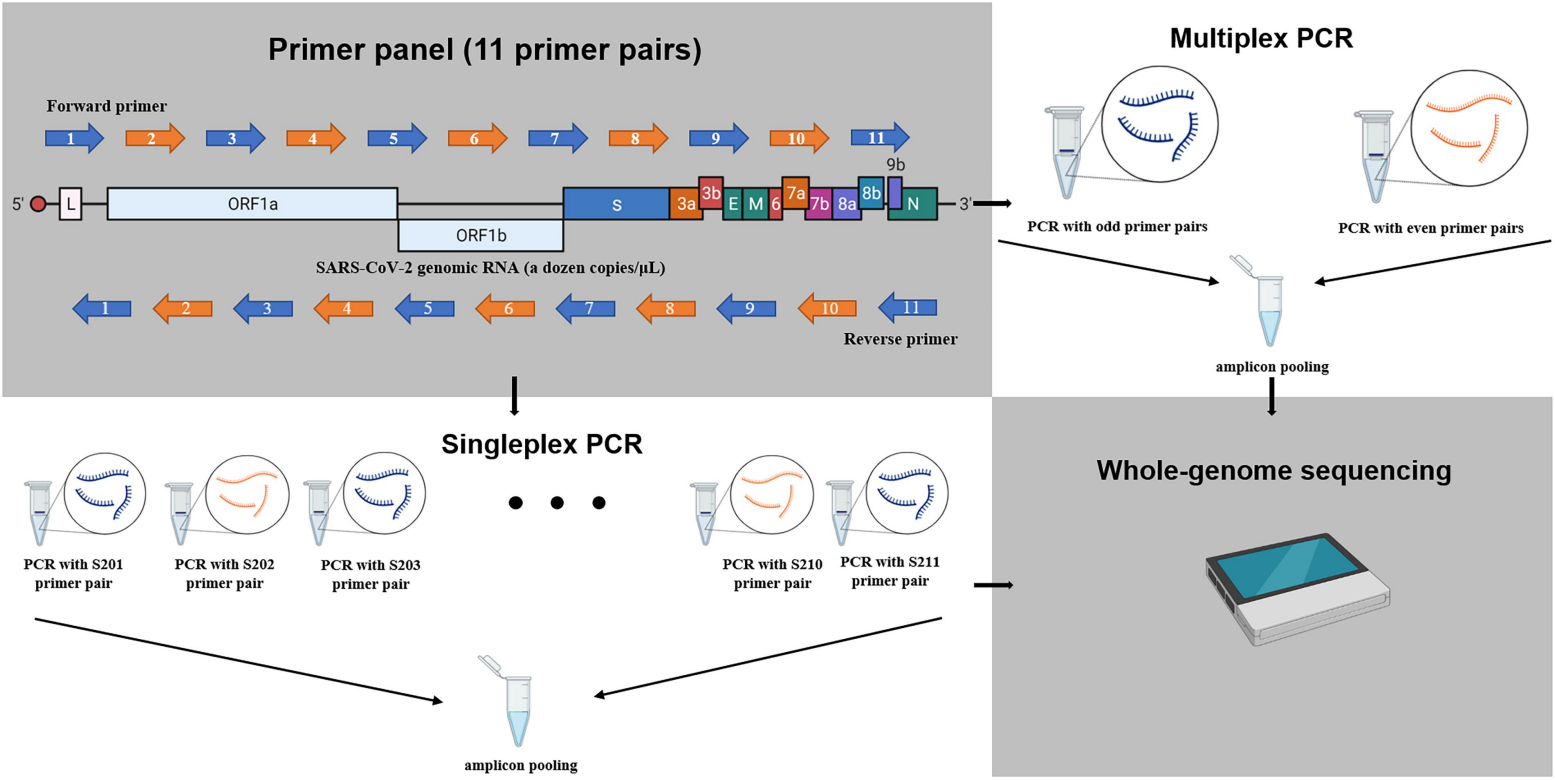
The schematic diagram for amplification of SARS-CoV-2 RNAs in this study. The whole genome of SARS-CoV-2 was amplified to 11 amplicons of approximately 3 kb using 11 primer panel. The blue and orange arrow indicates odd primer pair or even primer pair, respectively. Singleplex: the 11 amplicons were amplified with 11 individual reaction mixtures and pooled. Multiplex: the 11 amplicons were amplified with two reactions mixtures (six odd-numbered primer pairs and five even-numbered prime pairs) and then pooled. The schematic figure was drawn with Biorender.

The cDNAs of the clinical samples and the cultured samples were used as templates for the PCR amplification with 11 primer panels for the genome sequencing (Table 1). The amplification of the SARS-CoV-2 genome was done by 11 individual amplicon PCRs (singleplex) and by tiling PCR with two reaction mixtures (multiplex). The size of each amplicon was approximately 3 kb and confirmed with agarose gel electrophoresis (Figure 1). The singleplex reaction mixture had a volume of 50 μl and was done according to the manufacturer’s instructions (Supplementary Table S5). The PCR amplicons were then purified using the QIAquick PCR Purification Kit (QIAGEN, United States). After purification, the 11 fragments were pooled for sequencing (Figure 1).

**Table 1:** The sequence information of the primer panels for whole-genome sequencing of SARS-CoV-2.

Name	Primer pair	Sequence (5′ → 3′)	Product size (bp)
S201	7F	GGTTTATACCTTCCCAGGTA	3,187
	3194R	CTATCATCATCTAACCAATCTTC	
S202	2852F	GAACTCGGTACAGAAGTAA	2,887
	5739R	GGTAATTACCAGTGTACTCAC	
S203	5551F	TAAGGGTGTAGAAGCTGTTAT	3,194
	8745R	AAATCAGCATGTTTGTTAGCAA	
S204	8491F	GACATGTGCAACTACTAGAC	2,874
	11365R	CTCCTAGCACCATCATCATAC	
S205	11112F	TGGGTATTATTGCTATGTCTGC	2,969
	14081R	CACCGAAATCATACCAGTTAC	
S206	13025F	GCTGGTAATGCAACAGAAGTG	3,405
	16430R	CAATAATAGCTCATACCTCCTA	
S207	16025F	TAGCTATAGATGCTTACCCAC	2,926
	18951R	CTTTCTACAAGCCGCATTAATC	
S208	18712F	GCCTGTTGGCATCATTCTATTG	3,223
	21935R	TTCACAGACTTTAATAACAACATT	
S209	21528F	TTCTAGTGATGTTCTTGTTAAC	2,756
	24284R	CATTCTGTGTAACTCCAATACC	
S210	24055F	TGGCTTCATCAAACAATATGGT	3,294
	27349R	GTTGCTMTTCATCTAATTGAGA	
S211	27100F	TTGCTGCATACAGTCGCTAC	2,752
	29852R	TGTCATTCTCCTAAGAAGCT	

The multiplex PCR was done with odd-numbered primer pairs (S201, S203, S205, S207, S209, and S211) and even-numbered primer pairs (S202, S204, S206, S208, and S210). Both the odd and even reaction mixtures had a volume of 100 μl and were done according to the modified manufacturer’s instructions (Supplementary Table S5). After purification using the QIAquick PCR Purification Kit (QIAGEN, United States), both products from the odd and even mixtures were pooled for sequencing (Figure 1).

### MinION sequencing and assembly

The sequencing library for MinION (MK1B, Oxford Nanopore) was constructed using a ligation kit (SQK-LSK109, Oxford Nanopore) and barcoding kit (EXP-NBD104, Oxford Nanopore). MinION 1D amplicon sequencing was performed using the R9.4 Flow cell (FLO-MIN106, Oxford Nanopore) according to the manufacturer’s instructions. The quality of the sequencing reads was initially evaluated using the fastqc program ver. 0.11.8 (Babraham Bioinformatics, n.d.), and basecalling was performed using the Guppy basecaller ver.3.4.4 + a296acb (Oxford Nanopore) for an hour. The resultant fastq file was assembled using BWA-MEM version.0.7.12 (Li, 2013), and error correction was performed by the Pilon program version.1.24 (Walker et al., 2014). Reference mapping was done using SARS-CoV-2 (NC_045512.2, 376207.1, and OU327262) as a reference genome. The quality of the draft genome assemblies was checked and aligned using the Nextclade web application (Hadfield et al., 2018). The assembled contigs were further manually cured using the CodonCode aligner 8.0.2 (CodonCode Corporation; Li, 2013).

### iSeq100 sequencing and assembly

The second-strand cDNA was synthesized from the first-strand cDNA of the SARS-CoV-2 variants using the Invitrogen Second Strand cDNA Synthesis Kit (Thermo Fisher Scientific, United States) according to the manufacturer’s protocol. After synthesis of the second-strand cDNA, the residual RNA was removed using 100 U of RNaseI at room temperature for 5 min. The products were purified using DNA Clean and Concentrator Kits (Zymo Research, United States), and the purified DNAs were quantified using the QuantiFluor ds DNA System (Promega, United States) and Quantus™ Fluorometer, according to the manufacturer’s instructions. The sequencing libraries for iSeq100 were prepared using the Illumina DNA Prep Kit (Illumina, United States) which makes 300–350 bp DNA fragments, according to the manufacturer’s instructions. The concentration of the prepared libraries was determined using the KAPA Library Quantification Kit (Roche, Switzerland) with the LightCycler® 96 instrument (Roche, Switzerland) according to the manufacturer’s instructions. PhiX control v3 (Illumina, United States) was used as a quantification standard for the qPCR quantification. The final concentration of the pooled libraries was 100 pM, and a 5% PhiX control was added to the library. The pooled libraries were sequenced using iSeq100 (Illumina, United States). The assembly of the sequence reads was done using the CLC genomic workbench 20.0.4 (QIAGEN, United States) with the genome sequence of SARS-CoV-2 Wuhan-hu-1 (GenBank: NC_045512.2) and SARS-CoV-2 (OU327262) as a reference genome.

### Analysis of the sequence variance in the viral genomes from the clinical samples

The viral genome sequences from the clinical samples were used as queries for BLAST in the GISAID website, and the closest SARS-CoV-2 genome sequences were retrieved. The viral genomic sequences including hCoV-19/Wuhan/WIV04/2019 (GISAID: EPI_ISL_402124) and hCoV-19/bat/Yunnan/RaTG13/2013 (GISAID: EPI_ISL_402131) were aligned and classified using the Nextclade web application (Hadfield et al., 2018).

### Viral genome sequence deposition

The viral genome sequences from this study were deposited in GISAID and GenBank. The detailed information of the deposited sequences is listed in Supplementary Table S6.

## Results

### Quantification of RNA from the clinical samples and cultured SARS-CoV-2 strains

**Figure 2:**
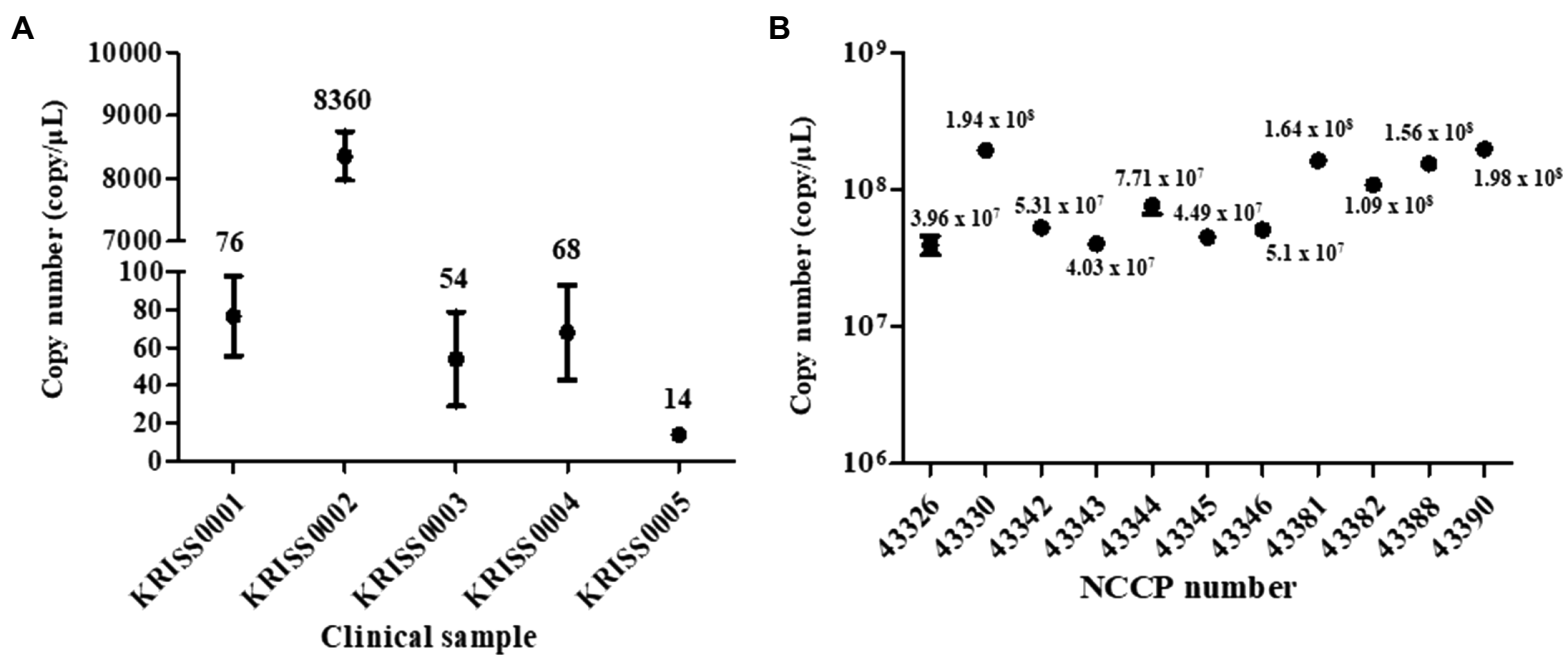
Quantification of SARS-CoV-2 RNA using a reverse transcription droplet digital PCR (RT-ddPCR). The copy numbers of SARS-CoV-2 RNA were measured RT-ddPCR assay using N gene targeting primer-probe sets. **(A)** The copy number of SARS-CoV-2 RNA in clinical samples. **(B)** The copy number of viral RNA in NCCP samples. The numbers of the above point and bars indicate mean copy numbers and standard deviation.

The measured concentrations of the RNA samples are listed in Supplementary Table S2. The standard curves of both the RT-qPCR and RT-ddPCR were linear indicating that the amplification efficiency of both assays was consistent (Figure 2; Supplementary Table S2; Supplementary Figure S1). The RNAs from the clinical samples were directly used as templates for the whole-genome amplification. The RNAs from the four cultured SARS-CoV-2 NCCP VOC variants were diluted to approximately a thousand, hundred, and 15 to 20 copies/μl and used as templates for the whole-genome amplification.

In the RT-qPCR results, the R^2^ value was nearly 1 (Supplementary Table S2). The concentration of the five clinical RNAs was 76, 8,360, 54, 68, and 14 copies/μl, respectively (Figure 2A). Moreover, the copy numbers of the RNAs from the strains NCCP VOC variants were determined as 1.64 × 10^8^ and 1.09 × 10^8^, 1.56 × 10^8^, and 1.98 × 10^8^, respectively (Figure 2B; Supplementary Table S2).

### Selection and validation of the universal primers for SARS-CoV-2

The designed primer candidates were screened using PCR amplification. The cDNAs synthesized from the two SARS-CoV-2 NCCP VOC variants were used for the screening. As a result, 11 primer panels that cover the whole-genome of SARS-CoV-2 were selected as the universal primer panel. The amplicons of the expected size were confirmed with agarose gel electrophoresis. The expected size of the amplicons was 2.7–3.4 kb, and the PCR products of the expected size were confirmed with both RNAs from NCCP VOC variants (Table 1; Supplementary Figure S2). The selected 11 primer panels were used for PCR amplification of the RNA from the other SARS-CoV-2 variants and clinical samples.

The universality of the selected primer panel was further validated with seven RNAs from different GISAID clade strains (S, L, V, G, GR, GH, and GV). The PCR reaction with the selected primer panel successfully produced the amplicons with the expected size from all the viral RNAs tested in this study (Supplementary Figure S2). Among these amplicons, the amplicons from the SARS-CoV-2 variant strains NCCP VOC variants were used as subjects for the sequencing.

### Genome sequencing of the SARS-CoV-2 variants

The genomes of four SARS-CoV-2 VOC variants were sequenced using iSeq100 and MinION. The double-stranded cDNAs of the four SARS-CoV-2 variants were sequenced using iSeq100, and approximately 10% of the sequence reads from iSeq100 were mapped to the reference sequences and covered the whole region of the SARS-CoV-2 genomes. The amplicons from the four SARS-CoV-2 variants were sequenced using MinION. The RNA concentrations for MinION sequencing were approximately 10, 10^2^, and 10^3^ copies/μl. The consensus sequence was determined based on the three complete genome sequences obtained by MinION with 10^2^ and 10^3^ copies/μl RNA and iSeq100. The assembly of the MinION sequencing samples with 156 to 198 copies/μl covered the whole region of the genomes. However, the coverages of NCCP 43381 and 43,382 with 15 to 20 copies/μl were 92.33% and 75.39%, respectively. Contrastively, the coverages NCCP 43388 and 43,390 with 15 to 20 copies/μl showed relatively complete sequences. These levels of LoD indicate that our amplicon-based sequencing methods can be applied to clinical samples with a low viral titer.

Compared to the genome sequence of iSeq100 sequencing, the consensus sequences of four VOC strains in MinION sequencing showed differences in the nucleotide positions (Supplementary Table S7). All the amino acid differences of the genomes sequenced in this study did not affect the variant classification results of the GISAID and Nextclade. The consensus genome sequences of NCCP 43381 showed four nucleotide differences according to the sequencing method and the RNA concentration, although all three consensus genome sequences of NCCP 43382 were identical (Supplementary Table S7). The consensus genome sequences of NCCP 43388 and 43,390 in MinION showed relatively more nucleotide differences compared to those of iSeq100. But all the mapped reads in these nucleotide position were showed both different nucleotides (Supplementary Tables S7, S8). For example, the nucleotide differences at position 23,608 were G (MinION)/A (iSeq100) in the genome sequence of NCCP 43381. However, the nucleotide of mapped reads at the positions was mixed with these two nucleotides (G/A), indicating both variants coexisted in the samples. The operational cost comparison of iSEQ100 and MinION sequencing showed that MinION sequencing has better cost-performance (Supplementary Table S9). Supplementary Table S9 showed the operational cost of MinION sequencing with six cultured viral RNA samples (amplicon-based sequencing) and that of iSeq100 sequencing with two cultured viral RNA samples (shotgun approach). Considering the high concentration of laboratory cultured virus and the depth of assembled sequences, the amplicon-based sequencing can be very efficient with low concentration samples.

### Genome sequencing of the clinical samples

The amplicons produced from both the singleplex and multiplex PCRs were pooled and sequenced using MinION. The trimmed quality of the sequencing raw reads had a median Phred score of 14 (Min q-score > 7). A single contig of full-length genome was successfully obtained from all five clinical samples. The genome sequence analysis from Nextclade is summarized in Supplementary Table S10. The MinION sequencing results of the multiplex and singleplex PCR products were both successful though the sequencing read depth was higher in the singleplex PCR than in the multiplex PCR due to a higher input amount of amplicons. There were five nucleotide substitutions in Wuhan-Hu-1 (NC045512; G5572T, G11083T, C14805T, C25487T, and G26144T) or one more (G11071T). These five nucleotide substitutions included four nonsynonymous substitutions (NS3-G251V, NS3-T32I, NSP3-M951I, and NSP6-L37F) and were identical in all five clinical samples. Strain hCoV-19/South Korea/KRISS0004/2020 (GISAID: EPI_ISL_3118363) had one more nonsynonymous substitution (NSP6-L33F). All other GISAID retrieved Korean viral genome sequences had four nucleotide substitutions (G5572T, G11083T, C14805T, and G26144T). Considering all GISAID retrieved United States viral genome sequences had three nucleotide substitutions (G11083T, C14805T, and G26144T) and the viral genome sequence from Spain had only two nucleotide substitutions (G11083T and G26144T), these viral genomes sequences might be the ancestral strains of these Korean strains. The results show that multiplex PCR is an applicable method for clinical samples.

## Discussion

### Quantification and sequencing LoD of the SARS-CoV-2 RNA genome

In this study, all RNAs of the NCCP strains and clinical samples were quantified by a Quantus fluorometer and RT-ddPCR. The quantification based on the fluorometer assay can estimate the total amount of RNA. As this assay does not require a primer-probe set to amplify specific genes, the quantification using a fluorometer is very convenient but there are some limitations. The fluorometer assay measures the concentration of the total RNA rather than that of RNA with specific sequences. On the other hand, RT-ddPCR can perform absolute quantification of RNA by counting each positive droplet without a standard curve and calibration (White et al., 2009; Lee et al., 2021; Park et al., 2021). Due to the presence of the host cell RNA, the estimated copy number of the fluorometer assays was greatly different from that of the RT-ddPCR assays, indicating that the exact assessment of the LoD requires a very accurate quantification such as RT-ddPCR assays.

The LoD of the amplicon-based sequencing with the four SARS-CoV-2 variants using singleplex PCR was estimated as 10 copies/μl which is similar to the LoD of the RT-qPCR assays. Although the genome sequences were not fully covered with 10 copies/μl of the NCCP 43381 and 43382, the absence of template can be statistically possible with a low concentration of viral RNA. Assuming that the mean copy number is 10 copies/μl with a standard deviation of 5 copies/μl and the mean follows a normal distribution, approximately 5% of the samples have no template. This absence of template suggests that the uncovered regions in the genome were due to the nature of the very low concentration rather than the limitation of the primer panels. This explanation is also supported by the results of the clinical samples. The lowest concentration of clinical samples was 14 copies/μl of SARS-CoV-2, and the assemblies from both the singleplex and multiplex PCRs covered the whole region of the genomes. Considering the very high Ct value (>35) of some clinical samples, our methods were able to sequence the whole-genome of any RT-qPCR positive samples. Although the Ct value of hCoV-19/South Korea/KRISS0005/2020 was undetermined by qPCR, the 14 copies/μl were quantified by ddPCR (Park et al., 2021). So, it can be sequencing based on amplicon like cultured viral RNA.

### Comparison of iSeq100 and MinION sequencing

The assembly of both iSeq100 and MinION was practically compared. The consensus sequences of NCCP 43882 were identical and those of NCCP 43881 showed only four different nucleotide positions. Although genome sequences of NCCP43388 and NCCP 43390 show relatively more differences in nucleotide positions, some of the mapped reads showed single-nucleotide variants (SNVs) in these positions (Supplementary Table S7). The iSeq100 assembly of NCCP 43381 showed SNVs in three of four sites except nucleotide position 17,616. The three sites were mixed with two nucleotides and one of these nucleotides was dominant in the MinION assembly (Supplementary Table S8). Although the dominant nucleotides in position 17,616 of MinION were the same as iSeq100 assembly, SNVs were observed in this site, suggesting bias from PCR amplification. The positions of nucleotide difference in NCCP 43388 and NCCP 43390 showed single nucleotides in iSeq100 assembly while two dominant nucleotides in MinION assembly. And each one of two dominant nucleotides in MinION assembly is the same as iSeq100 assembly. The reasons for polymorphism in MinION assembly were unclear but one of the possible reasons for these nucleotide differences can be laboratory mutation. According to the Nextclade analysis, the difference nucleotides of MinION were not private mutations which meant that the nucleotide differences were also observed in other strains. In the MinION assembly of NCCP 43388, both read with gaps and without gaps were shown. And these gaps also could be found in other SARS-CoV-2 genome sequences in public databases, indicating these mutations can occur naturally. These suggest that these nucleotides can be actual mutations rather than PCR bias or sequencing errors. Although the RNA templates for both iSeq100 and MinION sequencing were retrieved from the same viral strains, the genomic RNAs were extracted from different passages of viral culture. Considering all the SNVs were not private mutations, those SNVs observed in this study are likely to be cell-culture adaptive mutations frequently observed when inoculating a patient-originated virus into laboratory cell culturing conditions (Dargan et al., 2010; Oka et al., 2020). This suggests that the methods can detect minority variants in mixed ones. It is particularly important to diagnose the minority variant of SARS-CoV-2 due to the high mutation rate of this virus (Houldcroft et al., 2017). According to the results of this study, both MinION and iSeq100 enable the detection of minor variants among the virus population.

Considering the sequencing results of both iSeq100 and MinION, the accuracy of both methods was equal although some previous studies claimed that viral genome sequencing with MinION was not a reliable method unless the sequencing was replicated (Bull et al., 2020; World Health Organization, 2021). The viral genome sequencing through a shotgun approach using a short-read sequencer such as iSeq100 might be the best approach when the genomic information of the virus is not fully available. However, there are some drawbacks. Because this shotgun approach is not a target-specific approach, most of the sequence reads were non-target sequence reads. Though the viral sequencing using iSeq100 in this study was done with viral RNA using a high concentration, only 10% of the sequence reads were mapped to the reference sequence. Considering that the titer of the cultured virus was much higher than that of the clinical samples, the sequence reads from the shotgun approach with the clinical samples were mostly non-target sequence reads, and only a very small portion of the sequence reads can be used for the viral genome assembly. This is especially true with clinical samples having low viral titers. For this reason, amplicon-based genome sequencing of clinical samples can be more efficient if the genome sequence information is available.

### Analysis of the sequence variance in the clinical samples

Nextclade analysis showed that the nucleotide substitutions of the viral genome sequences in this study were not derived from PCR bias or sequencing error. Although there are no other strains with five substitutions like in the clinical samples sequenced in this study, these clinical samples were derived from different individual patients, indicating these substitutions actually occurred rather than resulting from an error. Four of the five nucleotide substitutions were found in other viral genomes from Korean clinical samples that first appeared on 17 February 2020, in GISAID. Currently, 138 strains with these four substitutions were uploaded in GISAID. The last strain with these substitutions was hCoV-19/South Korea/KCDC31-NCCP43342/2020 (GISAID: EPI_ISL_812962) which was collected on 28 July 2020. Most of these strains were collected from February to April in Korea, suggesting that the G5572T substitution occurred during their introduction to Korea. The strains from Spain and the United States had two (G11083T and G26144T) and three (G11083T, C14805T, and G26144T) nucleotide substitutions, respectively. These nucleotide substitutions were the same amino acid substitutions. Currently, the number of strains with these amino acid substitutions in GISAID is 6,929. The earliest strains with these amino acid substitutions were hCoV-19/Hangzhou/HZCDC0167/2020 (GISAID: EPI_ISL_421223) and hCoV-19/Italy/LAZ-INMI-SPL1/2020 (EPI_ISL_412974). These two strains had two identical nucleotide substitutions which suggest these two strains had the same origin. Most of the strains with these two amino acid substitutions were collected from January to August worldwide, and the last reported strains with these amino acid substitutions were collected in April 2021. All these findings indicate that the initial introduction of this SARS-CoV-2 lineage was done through Europe and the USA, and their spreading in Korea was successfully prevented.

### Amplicon-based genome sequencing of SARS-CoV-2

The ARTIC network multiplexed primer panels were used from the start of the COVID-19 pandemic, and the genome of SARS-CoV-2 can be sequenced with tilling PCR (Itokawa et al., 2020; Prado-Vivar et al., 2020; Tyson et al., 2020). However, the ARTIC primer panels consist of hundreds of primers and could have a potential vulnerability to the variations of the SARS-CoV-2 strains. Due to the nature of amplicon-based sequencing, mutations can occur in the target sequences of the primers, indicating more primers mean more mutations in the primer sites. The primer panels in this study consisted of only 11 primer pairs and potentially covered more SARS-CoV-2 variants than the panel with hundreds of primer pairs. The short amplicons of the ARTIC primer panels are very suitable for Illumina sequencers, but the short amplicons produce less nucleotide base output for long-read sequencers such as MinION. The amplicon size in this study was approximately 10-times larger than those of the ARTIC primer panels, and genome sequencing using long-read sequencers can be more efficient with longer amplicons.

The relatively inaccurate sequence reads of MinION sequencing have been pointed out as one of its major drawbacks for viral genome sequencing. However, MinION sequencing with our primer panels had an equivalent consensus sequence accuracy to that of the Illumina sequencer. The LoD of our primer panels was 15 to 20 copies/μl showing that our primer panels were enough sensitive for the sequencing of any clinical samples. The sequencing results of the individual clinical samples were identical except for one variant, and this also proved that the MinION sequencing with our primer panels can produce very accurate sequencing results from clinical samples.

## Conclusion

In this study, amplicon-based whole-genome sequencing of SARS-CoV-2 was established using MinION. Because the size of the amplicons in this study was longer than those of any other studies for amplicon-based whole-genome sequencing, the established methods can be very efficient SARS-CoV-2 genome sequencing methods for MinION. The primer panels consisted of only 11 primer panels and showed an excellent LoD. Whole-genome sequences were recovered from clinical samples containing only 15 to 20 copies/μl of SARS-CoV-2 RNA, and the sequencing results also showed that our method was very accurate and reliable for any RT-qPCR positive clinical samples.

## Data availability statement

The datasets presented in this study can be found in online repositories. The names of the repository/repositories and accession number(s) can be found in the article/Supplementary material.

## Author contributions

CP, KK, HY, and SK: conceptualization and writing—original draft. CP, KK, and DP: formal analysis, validation, and visualization. SK: funding acquisition. CP, KK, DP, and ZH: investigation. HY and SK: methodology, project administration, supervision, and writing—review and editing. EP, C-SL, MR, HY, and SK: resources. All authors contributed to the article and approved the submitted version.

## Funding

This work was supported by the National Research Council of Science and Technology grant by the Ministry of Science and ICT (grant no. CRC-16-01-KRICT). This work was also supported by the “Establishment of measurement standards for microbiology,” grant number GP2021-0003-08, “Development of Rapid Diagnosis of Rift Valley Fever Virus based on Whole Genome Analysis,” grant number HI20C0558, and “Development of Rift valley fever virus RNA reference materials,” grant number 20015205 funded by the Korea Research Institute of Standards and Science, the Ministry of Health and Welfare, and Ministry of Trade, Industry and Energy, respectively.

## Conflict of interest

The authors declare that the research was conducted in the absence of any commercial or financial relationships that could be construed as a potential conflict of interest.

The patent of the primer panel for the whole-genome sequencing is pending in Korea (10-2020-0111836).

## Publisher’s note

All claims expressed in this article are solely those of the authors and do not necessarily represent those of their affiliated organizations, or those of the publisher, the editors and the reviewers. Any product that may be evaluated in this article, or claim that may be made by its manufacturer, is not guaranteed or endorsed by the publisher.

## Supplementary material

SUPPLEMENTARY FIGURE S1 The RT-qPCR of cultured SARS-CoV-2 variant. The RT-qPCR assay using N gene targeting primer-probe set showed the linearity of standard curves.

SUPPLEMENTARY FIGURE S2 Agarose gel electrophoresis of eleven amplicons from SARS-CoV-2 RNA variants.
