# Supplementary figures and images for: Rapid and sensitive amplicon-based genome sequencing of SARS-CoV-2
Source: Front Microbiol. 2022 Aug 17;13:876085. doi: 10.3389/fmicb.2022.876085 (PMC9428490; doi:10.3389/fmicb.2022.876085)

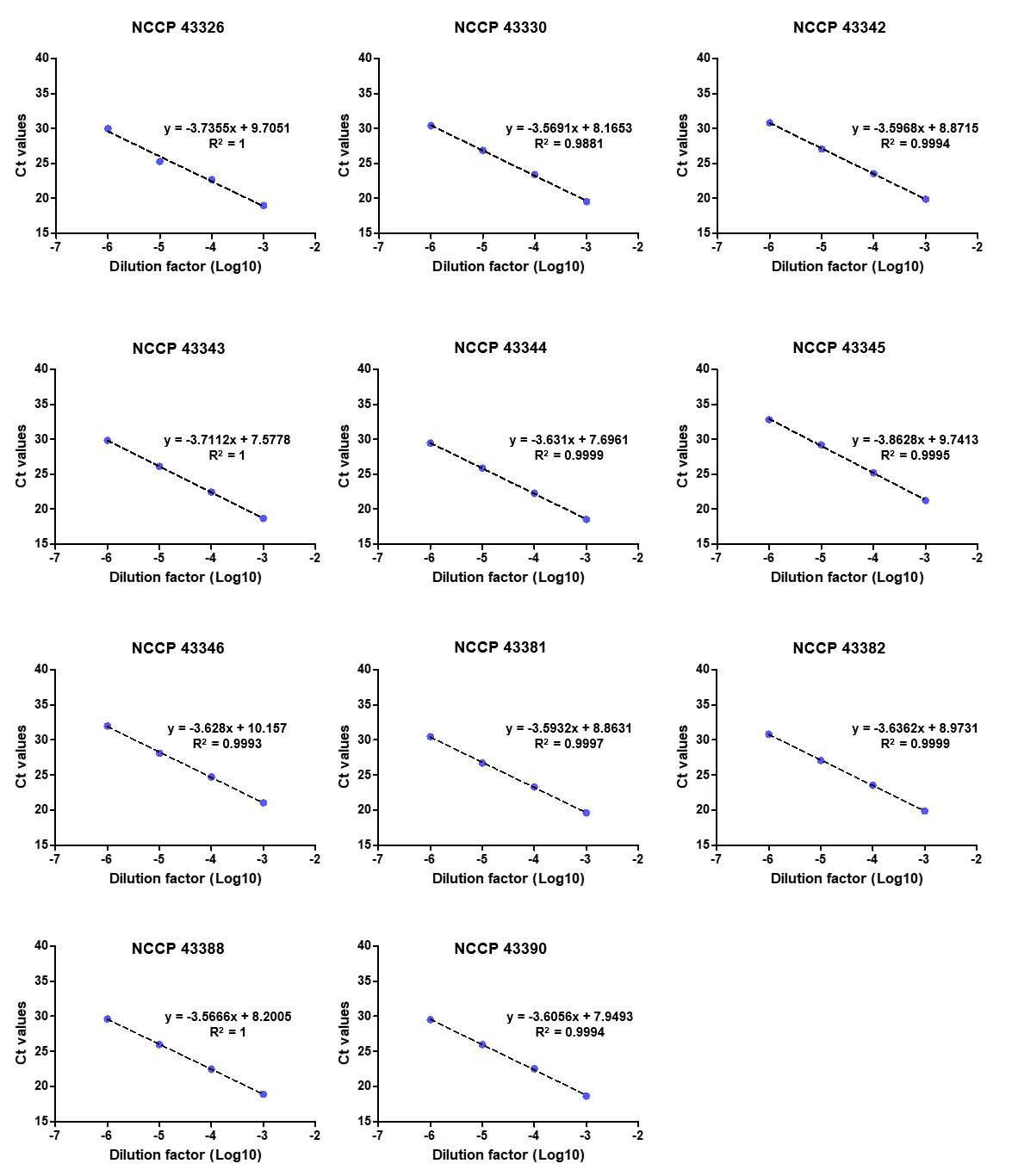

Supplement: SUPPLEMENTARY FIGURE S1 — The RT-qPCR of cultured SARS-CoV-2 variant. The RT-qPCR assay using N gene targeting primer-probe set showed the linearity of standard curves. [file Image_1.TIF]

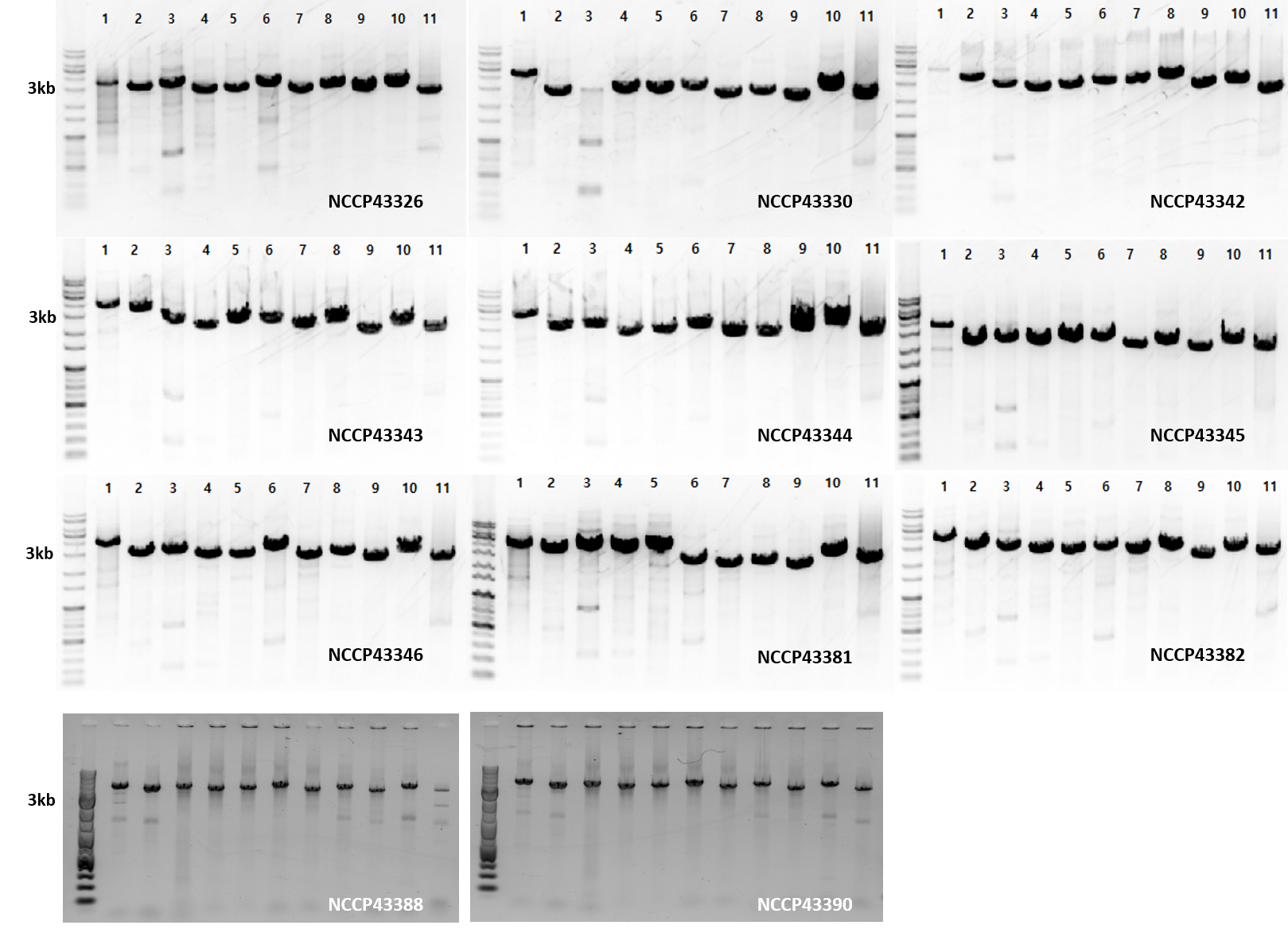

Supplement: SUPPLEMENTARY FIGURE S2 — Agarose gel electrophoresis of eleven amplicons from SARS-CoV-2 RNA variants. [file Image_2.TIF]
